# Cognitive behavioral interventions for depression and anxiety in adults with neurological disorders: a systematic review and meta-analysis

**DOI:** 10.1017/S0033291724001995

**Published:** 2024-09

**Authors:** Milena Gandy, Thomas Woldhuis, Wendy Wu, Marette Youssef, Madelyne A. Bisby, Blake F. Dear, Andreea I. Heriseanu, Amelia J. Scott

**Affiliations:** School of Psychological Sciences, Macquarie University, Sydney, Australia

**Keywords:** acceptance and commitment therapy, cbt, mood, neurological disease, neuropsychiatry, psychotherapy

## Abstract

We examined the efficacy of cognitive and behavioral interventions for improving symptoms of depression and anxiety in adults with neurological disorders. A pre-registered systematic search of Cochrane Central Register of Controlled Trials, MEDLINE, PsycINFO, Embase, and Neurobite was performed from inception to May 2024. Randomized controlled trials (RCTs) which examined the efficacy of cognitive and behavioral interventions in treating depression and/or anxiety among adults with neurological disorders were included. Estimates were pooled using a random-effects meta-analysis. Subgroup analyses and meta-regression were performed on categorical and continuous moderators, respectively. Main outcomes were pre- and post-intervention depression and anxiety symptom scores, as reported using standardized measures. Fifty-four RCTs involving 5372 participants with 11 neurological disorders (including multiple sclerosis, epilepsy, stroke) were included. The overall effect of interventions yielded significant improvements in both depression (57 arms, Hedges' *g* = 0.45, 95% confidence interval [CI] 0.35–0.54) and anxiety symptoms (29 arms, *g* = 0.38, 95% CI 0.29–0.48), compared to controls. Efficacy was greater in studies which employed a minimum baseline symptom severity inclusion criterion for both outcomes, and greater in trials using inactive controls for depression only. There was also evidence of differential efficacy of interventions across the neurological disorder types and the outcome measure used. Risk of bias, intervention delivery mode, intervention tailoring for neurological disorders, sample size, and study year did not moderate effects. Cognitive and behavioral interventions yield small-to-moderate improvements in symptoms of both depression and anxiety in adults with a range of neurological disorders.

## Background

Depression and anxiety are prevalent among people with neurological disorders (NDs) (Alsaadi et al., 2019; Hesdorffer, 2016; Whitney, Shapiro, Warschausky, Hurvitz, & Peterson, 2019) and impact quality of life and prognosis (Hesdorffer, 2016; Prisnie et al., 2018). Although there are distinct etiological and pathological factors associated with these comorbidities among discrete ND types, they share several psychosocial risk factors. These include challenges associated with a lack of independence and sleep disturbances, and vulnerabilities associated with coping styles and cognitive difficulties (Cieza et al., 2015; Gandy et al., 2021). Notably, enhancement of rehabilitation and support services to address prevalent psychosocial challenges and mental health issues across NDs has recently been highlighted as a target within the World Health Organization Intersectoral Global Action Plan (IGAP) on epilepsy and other NDs (World Health Organisation, 2023).

Cognitive- and behavior-based therapies are common psychological interventions for depression and anxiety in the general population, and have been recommended in practice guidelines to manage mental health difficulties in NDs including epilepsy (Mula et al., 2022), multiple sclerosis (MS) (Minden et al., 2014), and traumatic brain injury (TBI) (Marshall, Bayley, McCullagh, Velikonja, & Berrigan, 2012). These interventions aim to promote adaptive coping skills and address unhelpful patterns in thinking styles and behaviors known to exacerbate poor mental health. Previous meta-analyses have identified beneficial treatment effects of these interventions in specific ND subgroups (Ghielen et al., 2019; Hind et al., 2014; Wang et al., 2018). However, these meta-analyses are restricted by the availability of limited numbers of disorder-specific clinical trials with small sample sizes (ks = 7 [Hind et al., 2014] to 23 [Wang et al., 2018]), precluding firm conclusions around intervention efficacy and potentially informative moderator analyses. Moreover, meta-analyses rarely explore psychological interventions for managing both depression and anxiety, despite their frequent coexistence across NDs (Alsaadi et al., 2019). This gap is notable given the rise in transdiagnostic interventions, which aim to address both sets of symptoms (Dalgleish, Black, Johnston, & Bevan, 2020). Finally, previous meta-analyses have not included clinical trials from the past five years (Barua et al., 2024; Ghielen et al., 2019; Hind et al., 2014; Wang et al., 2018).

Overall, the efficacy of cognitive and behavioral interventions in NDs remains unclear, including whether the efficacy of these interventions differ depending on important sample, study design, and intervention-related factors. These comparisons are important given some previous equivocal findings on the efficacy of these interventions for adults with common NDs such as epilepsy (Noble, Reilly, Temple, & Fisher, 2018) and MS (Sesel, Sharpe, & Naismith, 2018). Moreover, no meta-analysis has examined nor compared treatment effects of cognitive and behavioral interventions across NDs, despite broad public health targets to improve psychosocial care across NDs (World Health Organisation, 2023). Additionally, while tailoring interventions for NDs, and offering remote treatment options have been suggested (Gallagher, McLeod, & McMillan, 2019; Gandy, 2020, 2023), the impact of these factors on outcomes remains unclear. This is also the case for study related factors known to influence treatment effects including the type of control and outcome measures used, baseline symptom severity, and risk of bias (Gandy et al., 2022; Scott et al., 2023).

These analyses and findings may inform clinical care and referral decisions for adults with NDs and may assist the refinement of interventions in this area. Furthermore, efficacy benchmarks can inform future research directions, including sample size and power calculations for clinical trials, which are often done arbitrarily.

The primary aim of this systematic review and meta-analysis was to examine the efficacy of cognitive and behavioral psychological interventions for improving symptoms of depression and/or anxiety symptoms in adults with NDs. Potential study, sample, and intervention moderators of efficacy were also investigated to examine the robustness of clinical effects.

## Methods

### Registration

This study was completed in compliance with the Preferred Reporting Items for Systematic Review and Meta-Analyses (PRISMA) Checklist (Moher et al., 2015). The protocol was prospectively registered on PROSPERO (ID: CRD42022351547) and underwent one amendment to expand inclusion criteria to Functional Neurological Disorder (FND) as these patients are often managed within neurological care settings.

### Eligibility criteria

Studies were included if they (1) implemented a psychological intervention based on cognitive and behavioral principles, (2) targeted depression and/or anxiety symptoms and reported pre- and post-intervention outcomes of either using a standardized measure, (3) adopted a randomized controlled trial (RCT) design comparing the intervention against a control group, with at least 20 participants in each arm at randomization to minimize risk of bias associated with including studies with small samples (Ioannidis, 2005; Simmons, Nelson, & Simonsohn, 2011), and (4) recruited adult participants, with (5) a ND as defined by the National Institute of NDs and Stroke (NINDS). Cognitive and behavioral interventions were defined as those which emphasize relationships between thoughts, feelings, and behaviors that impact psychological wellbeing and teach cognitive and/or behavioral-based skills to improve these relationships, consistent with previous meta-analyses (Gandy et al., 2022). No restrictions were placed on the type of control group used, study setting, or mode of intervention delivery.

Studies were excluded if the psychological intervention did not primarily target mental health (e.g. pain or sleep) and the unique effects of the psychological component could not be isolated from a multimodal intervention, if no control group was included and if participants had NDs not classified by the NINDS or not primarily managed within neurological care settings (e.g. neurodevelopmental and sleep disorders, or purely symptom-based conditions including chronic pain). These criteria are largely consistent with previous reviews of anti-depressant medications for NDs (Price et al., 2011).

### Search strategy

Electronic databases Cochrane Central Register of Controlled Trials (CENTRAL), MEDLINE, PsycINFO, Embase and Neurobite were searched from inception to May 2024 with no language restrictions. The search strategy was developed with consultation from an expert librarian and included terms related to NDs, depression and anxiety, cognitive and behavioral interventions, and RCTs. See online Supplementary Method 1 for the full search strategy.

### Study selection

One author (T.W.) screened all titles and abstracts using Rayyan (Ouzzani, Hammady, Fedorowicz, & Elmagarmid, 2016), with a random 20% blind reviewed by a second author (M.G.) to verify the accuracy of the screening process. All full text articles were independently assessed by two authors (M.G. M.Y., T.W., or W.W.), with 90% agreement (*κ* = 0.76). Disagreements were resolved following discussion.

### Data extraction

Data from each study was independently double extracted by at least two reviewers (T.W., M.B., A.S., A.H., W.W) using a standardized data extraction sheet. Where studies implemented multiple eligible interventions, data from each intervention arm were extracted and control participant numbers were divided equally to avoid unit-of analyses error, consistent with Cochrane recommendations (Higgins et al., 2019). At least two attempted contacts were made with authors of 16 studies which were eligible but omitted data required for the meta-analysis; two authors were not contactable. Of the 14 successfully contacted, eight responded (57% response rate) with six of these providing data which was used in the analyses.

### Risk of bias

Risk of bias was assessed using version 2 of the Cochrane Collaboration's Risk of Bias assessment tool (RoB 2) (Higgins et al., 2011). The tool covers common areas of bias in RCTs that arise from five different RCT processes, including randomisation, deviations from the intended intervention, handling missing data, outcome measurement, and reporting of results. Based on study reporting they were rated as having high risk, low risk, or some concerns. We omitted items relating to concealment of intervention allocation to participants, as this is accepted practice in meta-analyses of psychotherapy RCTs (Munder & Barth, 2018) given the inherent inability to blind participants to intervention.

### Study bias

Study bias was assessed by visual inspection of funnel plots and Egger's test. Funnel plots which appear asymmetric and significant Egger's tests are each suggestive of small study effects. Trim-and-fill analysis was used to estimate the number of unpublished studies and provide an adjusted effect estimate if they were included (Duval & Tweedie, 2000).

### Data analysis

Statistical procedures were performed using Comprehensive Meta-Analysis (CMA), version 3.0 (Borenstein, Hedges, Higgins, & Rothstein, 2013). Meta-analyses used random effects models, assuming effect sizes vary due to within-study and between-study differences. Data were entered into CMA as pre-intervention and post-intervention means and s.d.s for treatment and control groups, or as mean differences within each arm and their s.d.s. CMA calculated Hedges' *g* effect sizes, representing the standardized mean difference corrected for small sample sizes. We interpreted *g* values of 0.2, 0.5, and 0.8 to indicate small, moderate, and large effects, respectively (Deeks, Higgins, & Altman, 2019). Heterogeneity between studies was assessed using the *I*^2^ statistic, with values of 40% or below indicating low heterogeneity, between 41% and 60% indicating moderate heterogeneity, and above 60% indicating substantial heterogeneity (Deeks et al., 2019). Tau squared (*t*^2^) was reported as a measure of between-study variance for each effect. Outliers were defined as studies with 95% confidence intervals (CIs) that do not overlap with those for the pooled effect (Harrer, Cuijpers, Furukawa, & Ebert, 2021). The influence of outliers on the pooled effect was examined using the ‘one study removed’ procedure (Harrer et al., 2021; Viechtbauer & Cheung, 2010).

### Moderator analyses

Moderator analyses were conducted to determine whether intervention efficacy was influenced by study, sample, and intervention characteristics (alpha set at 0.05). Moderators were only analyzed if there were at least three eligible comparators. Subgroup analyses using mixed effect models were performed for categorical variables of interest, including ND type (e.g. MS, epilepsy, PD, stroke, TBI, and migraine), whether the intervention was tailored to NDs (i.e. tailored *v.* untailored), mode of delivery (i.e. face-to-face, teletherapy, or digital), control group type (active *v.* inactive), whether baseline symptom severity was assessed as a recruitment inclusion criterion (i.e. required diagnosis or symptoms above cut-off or not), type of outcome measure used and risk of bias. Meta-regression was performed to examine the effect of continuous variables, including sample size and year of publication.

## Results

### Study selection results

**Figure 1.**
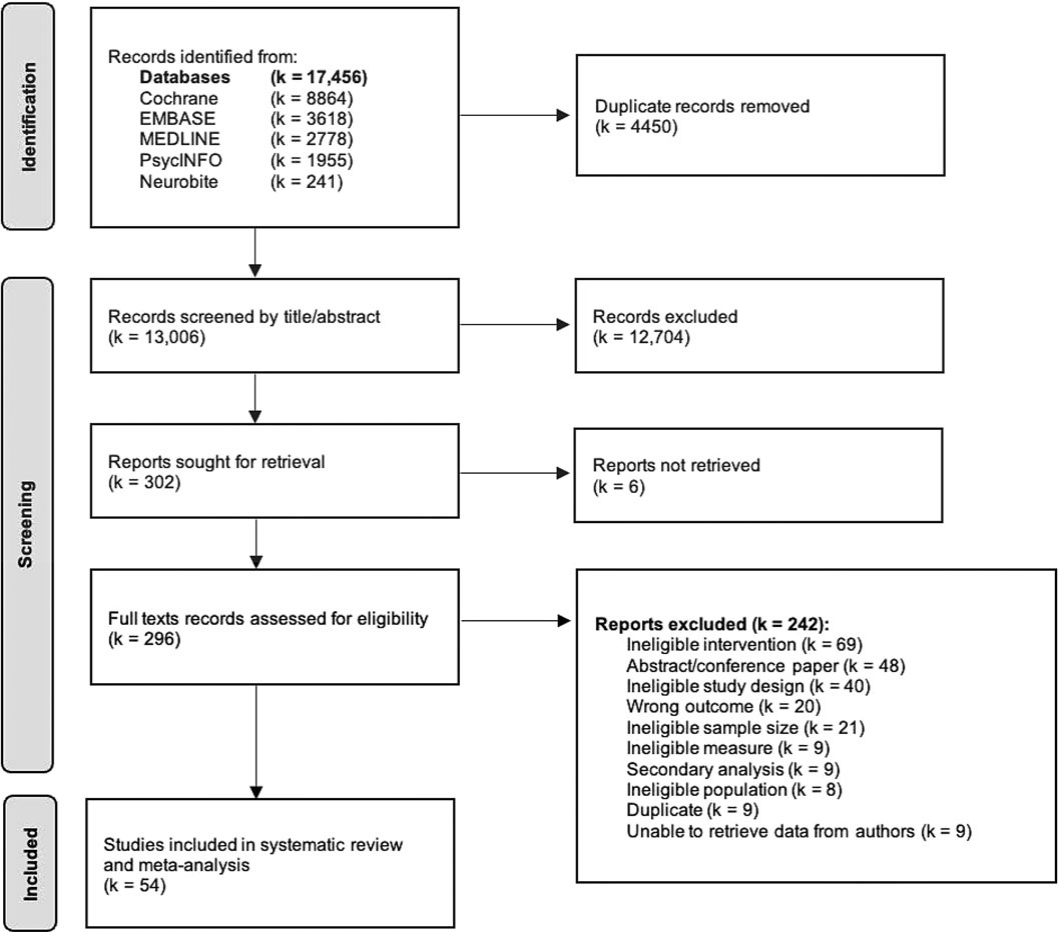
Prisma flow chart.

After records were retrieved and duplicates removed, 13 006 titles and abstracts were screened for eligibility. Of the 296 full texts articles reviewed, 54 met the inclusion criteria and were included in the analyses. A flow chart of the selection process is available in Fig. 1.

### Study characteristics

Study characteristics are summarized in Table 1 and online Supplementary Table S1. A reference list of included studies is available in online Supplementary References. A total of 5372 participants were included. Mean age ranged from 29.4 to 84.1 years and approximately half (57%) were female. Race/ethnicity was reported in 18 studies (52%), mostly from the USA (*k* = 14). Among these, participants were predominantly White/Caucasian (~86%, *k* = 14), followed by Black/African American (~6%; *k* = 10) and Hispanic/Latino (~6%; *k* = 9). While most studies (*k* = 43; 80%) reported on the educational attainment of their sample, there was considerable variability in reporting method that precludes meaningful synthesis. The most common NDs were MS (*k* = 12; 22%), epilepsy (*k* = 9; 17%), stroke (*k* = 10; 17%), Parkinson's Disease (PD; *k* = 7, 13%), and TBI (*k* = 6, 11%).

**Table 1.** Study characteristics

Study/Country	N	ND	Mean age, yrs (s.d.)	Female %	Mean DD, yrs	Int. type	Tailored	Int. delivery	Control group	Severity criterion	Outcome measures	Drop-out %: Int, C
A'Campo, Wekking, Spliethoff-Kamminga, Le Cessie, and Roos (2010)/NL	57	PD	64.95 (8.98)	45.3	5.73	CBT	Y	F2F; group	Inactive (UC)	N	SDS	8.6, 0.0
Assonov (2021)/UK	70	TBI	46.44 (7.67)	2.86	NR	CBT	Y	F2F; individual	Inactive (TAU)	N	HADS-D, HADS-A	0.0, 0.0
Bahrani, Zargar, Yousefipour, and Akbari (2017)/IR	47	MS	36.38 (6.57)	100	7.04	MiCBT	N	F2F; group	Inactive (TAU)	N	DASS21-D, DASS21-A	17.9, 14.3
Bailey, Stevens, LaRocca, and Scogin (2017)/US	51	Dementia	84.14 (8.37)	90.1	NR	BT	Y	F2F; group	Inactive (TAU)	Y	GDS	NR
Bédard et al. (2014)/CA	76	TBI	46.46 (13.41)	55.3	NR	MBCT	Y	F2F; group	Inactive (TAU)	Y	BDI-II	33.3, 20.8
Bell et al. (2017)/US	356	TBI	29.35 (7.23)	6.74	NR	PST	Y	Teletherapy; individual	Active (Edu)	N	PHQ-9	29.2, 11.2
Belleville et al. (2018)/CA	87	MCI	72.61 (6.58)	24.3	NR	CBT	N	F2F; group	Inactive (TAU)	N	GDS, GAI	0.0, 0.0
Boele et al. (2018)/NL	88	Glioma	44.88 (11.97)	57.8	NR	PST	Y	Digital-guided; individual	Inactive (WLC)	Y	CES-D	51.1, 20.5
Boeschoten et al. (2017)/NL	171	MS	48.90 (10.5)	80.1	11.20	PST	Y	Digital-guided; individual	Inactive (WLC)	Y	BDI-II, HADS-A	12.9, 9.3
Bogosian et al. (2022)/UK	60	PD	60.87 (10.11)	50	5.83	MBCT	Y	Digital-guided; group	Inactive (WLC)	N	HADS-D, HADS-A	30.0, 3.3
Bromberg et al. (2012)/US	185	Migraine	42.62 (11.5)	89.19	2.47	CBT	Y	Digital-unguided; individual	Inactive (TAU)	N	DASS21-D, DASS21-A	15.7, 5.3
Ciechanowski et al. (2010)/US	80	Epilepsy	43.90 (11)	52.5	NR	PST	Y	F2F; individual	Inactive (TAU)	Y	HSCL-20	20.0, 17.5
Dindo et al. (2020)/US	134	Migraine	35.76 (13.89)	82.5	NR	ACT	Y	F2F; group	Active (Edu)	Y	HAM-D, SIGH-A	29.6, 29.2
Dobkin et al. (2021)/US	90	PD	66.84 (8.65)	0	5.42	CBT	Y	Digital-guided; individual	Inactive (TAU)	Y	HAM-D, HAM-A	4.4, 4.4
Dobkin et al. (2020)/US	72	PD	65.22 (9.63)	51.39	6.33	CBT	Y	Teletherapy; individual	Inactive (TAU)	Y	HAM-D, HAM-A	10.8, 14.3
Dobkin et al. (2011)/US	80	PD	64.56 (10.53)	40	6.34	CBT	Y	F2F; individual	Inactive (TAU)	Y	HAM-D, HAM-A	12.2, 7.7
Ehde et al. (2015)/US	163	MS	52.19 (10.08)	87.09	NR	CBT	Y	Teletherapy; individual	Active (Edu)	Y	PHQ9	14.7, 8.0
Exner et al. (2022)/DE	56	ABI	45.65 (11.04)	46.4	2.31	CBT	Y	F2F; individual	Inactive (WLC)	Y	CES-D Short	0.0, 13.8
Fann et al. (2015)/US	100	TBI	45.80 (13.3)	37	3.33	CBT	Y	Mix; individual	Inactive (TAU)	Y	HAM-D	10.3, 14.3
Feng et al. (2022)/CN	95	Epilepsy	30.87 (5.94)	56.8	NR	CBT	Y	F2F; group	NR	N	HAM-D, SAS	2.1, 3.9
Fischer et al. (2015)/DE	90	MS	45.28 (11.58)	77.8	8.30	CBT	N	Digital-unguided; individual	Inactive (WLC)	Y	BDI	2.2, 0.0
Fraser et al. (2015)/US	78	Epilepsy	45.20 (12.5)	55	NR	PsychoEd	Y	F2F; group	Inactive (WLC)	N	PHQ9, GAD7	7.3, 4.8
Gandy et al. (2023)/AU	215	Mixed (MS, epilepsy, PD, ABI)^a^	53.00 (12.46)	79.1	11.48	CBT	Y	Digital-guided; individual	Inactive (WLC)	N	PHQ9, GAD7	7.2, 2.9
Gandy et al. (2014)/AU	45	Epilepsy	39.33 (12.52)	64.4	13.33	CBT	Y	F2F; individual	Inactive (WLC)	N	HADS-D, HADS-A	35.5, 10.7
Gold et al. (2023)/DE, US^b^	262	Multiple Sclerosis	46.95 (11.7)	76.34	10.70	CBT	Y	Digital-guided/unguided^b^; individual	Inactive (TAU)	Y	BDI-II	20.8, 16.5, 16.1
Graziano, Calandri, Borghi, and Bonino (2014)/IT	82	MS	40.50 (9.4)	64	7.90	CBT	Y	F2F; group	Active (Edu)	N	CES-D	12.2, 17.1
Hum et al. (2019)/CA	44	Epilepsy	37.05 (2.71)	70.9	NR	MiCBT	Y	Teletherapy; group	Active (Edu)	N	QIDS	31.0, 17.2
Johnson et al. (2020)/US	101	Epilepsy	42.90 (13.9)	62	NR	Psycho-ed	Y	Mix; group	Inactive (WLC)	N	PHQ9, GAD7	8.2, 3.8
Kirkness et al. (2017)/US^b^	91	Stroke	60.30 (NR)	49.97	NR	CBT	Y	Teletherapy/F2F^b^; individual	Inactive (UC)	Y	HAM-D	8.1, 8.6, 10.1
Kraepelien et al. (2020)/SE	77	PD	66.00 (9.12)	61	NR	CBT	Y	Digital-guided; individual	Inactive (WLC)	N	HADS-D, HADS-A	10.5, 2.6
Lin et al. (2023)/HK	171	MCI	69.13 (8.14)	87.7	NR	Mixed (CBT, PST, DBT, PsychoEd)	Y	Mix; group	Active (Edu)	N	GDS-SF	18.6, 12.9
Lincoln et al. (2011)/UK	151	MS	46.07 (10.86)	NR	9.88	CBT	Y	F2F; group	Inactive (WLC)	Y	HADS-D, HADS-A	15.3, 11.4
Liu et al. (2023)/CN	139	Stroke	61.9 (10.36)	34.5	NR	ACT	Y	F2F; group	Inactive (UC)	Y	HAM-D	0.0, 1.4
Majumdar et al. (2019)/UK	53	Stroke	62.70 (13.9)	39.6	4.17	ACT	Y	F2F; group	Inactive (TAU)	N	PHQ9, GAD7	3.8,14.8
Martin et al. (2015)/AU	66	Migraine, headache	40.64 (13.41)	74.24	19.45	CBT	Y	F2F; individual	Inactive (TAU)	Y	BDI-II, BAI	41.7, 16.7
Meyer et al. (2019)/DE	200	Epilepsy	40.30 (13.12)	63.5	NR	CBT	Y	Digital-unguided; individual	Inactive (TAU)	N	PHQ9, GAD7	27.0, 19.0
Migliorini, Sinclair, Brown, Tonge, and New (2016)/AU	48	Spinal cord injury	49.75 (12.67)	28.8	14.96	CBT + PP + MF	Y	Digital-guidance optional; individual	Inactive (WLC)	Y	DASS21-D, DASS21-A	32.4, 0.0
Mohr et al. (2005)/US	127	MS	47.96 (9.85)	77.17	11.23	CBT	N	Teletherapy; individual	Active (SEFT)	Y	BDI-II	3.2, 4.6
Moonen et al. (2021)/NL	48	PD	63.30 (7.80)	52	6.10	CBT	N	F2F; individual	Inactive (WLC + Monitor)	Y	HDRS, HARS	8.3, 8.3
Nazari, Aligholipour, and Sadeghi (2020) [a]/IR	64	MS	35.13 (5.28)	100	4.80	UP	Y	F2F; group	Active (TAU + SS)	Y	HADS-D, HADS-A	6.3, 12.5
Nazari, Sadeghi, Ghadampour, and Mirzaeefar (2020) [b]/IR	70	MS	35.30 (3.01)	61.4	3.31	UP	Y	F2F; group	Active (TAU + PsychoEd)	Y	HADS-D, HADS-A	20.0, 34.3
Niu et al. (2022)/CN	80	Stroke	63.15 (11.86)	34.61	NR	ACT	Y	F2Fl; group	Inactive (TAU)	N	HAM-D	5.8, 7.7
Pahlavanzadeh, Abbasi, and Alimohammadi (2017)/IR	70	MS	NR	100	NR	CBT	Y	F2F; group	Active (Group discussion + TAU)	Y	DASS42-D, DASS42-A	NR
Ponsford et al. (2016)/AU^b^	75	TBI	42.24 (14.45)	26.7	3.73	CBT + MI/CBT + NDC^b^	Y	F2F; individual	Inactive (WLC)	Y	DASS42-D, HADS-A	42.3, 30.8, 21.7
Potter, Brown, and Fleminger (2016)/UK	45	TBI	41.40 (11.60)	46	3.25	CBT	Y	F2F; individual	Inactive (WLC)	N	HADS-D, HADS-A	3.8, 0.0
Sadeghi-Bahmani et al. (2022)/IR	51	MS	38.68 (10.28)	8.16	NR	ACT	N	F2F; group	Inactive (WLC)	Y	BDI-FS	0.0, 3.8
Schröder et al. (2014)/DE	78	Epilepsy	37.59 (11.20)	75.64	NR	CBT + MF + ACC	N	Digital-unguided; individual	Inactive (WLC)	Y	BDI-I	34.2, 20.0
Simshäuser et al. (2022)/DE	54	Migraine	45.25 (10.54)	88.9	NR	MBCT	Y	F2F; group	Inactive (WLC)	N	HADS-D, HADS-A	25.9, 25.9
Spruill et al. (2021)/US	72	Epilepsy	43.30 (11.30)	70.8	25.50	MBCT	Y	Teletherapy; group	Inactive (TAU)	Y	PHQ-9	2.8, 11.1
Sun, Xu, Zhang, Zhou, and Lv (2022)/CN	65	Stroke	62.03 (9.07)	43.09	<0.25	BAT	N	F2F; individual	Active (TAU + Rehab + PsychoEd)	Y	CES-D	5.7, 8.6
Thomas et al. (2019)/UK	48	Stroke	65.60 (13.60)	39.6	NR	BAT	Y	F2F; individual	Inactive (TAU)	Y	PHQ-9	30.8, 8.7
Thomas, Walker, Macniven, Haworth, and Lincoln (2012)/UK	105	Stroke (Aphasia)	67.00 (13.54)	37	NR	BAT	Y	F2F; individual	Inactive (TAU)	Y	SAD-Q	19.6, 11.1
Visser et al. (2016)/NL	166	Stroke	53.06 (10.20)	47	NR	PST	Y	F2F; group	Inactive (UC)	N	CES-D	11.4, 0.0
Wang, Li, Wang, and Lv (2020)/CN	134	Stroke	59.93 (10.66)	53.73	NR	MBCT	N	F2F; group	Active (Stress management, Edu)	N	CES-D	33.7, 22.8

ABI, acquired brain injury; ACC, acceptance; ACT, acceptance and commitment therapy; AU, Australia; BAT, behavioural activation therapy; BDI, beck depression inventory; C, control; CA, Canada; CBT, cognitive behaviour therapy; CES-D, centre for epidemiological studies depression; CN, China; DASS, depression anxiety stress scales; DBT, dialectal behaviour therapy; DD, disease duration; DE, Germany; Edu, education; F2F, face-to-face; GAD, general anxiety disorder; GAI, geriatric anxiety inventory; GDS, geriatric depression scale; HADS, hospital anxiety depression scale; HAM-A/HARS, hamilton anxiety rating scale; HAM-D/HDRS, hamilton depression rating scale; H, Hong Kong; HSCL, hopkins symptom checklist; Int., intervention; IR, Iran; IT, Italy; MBCT, mindfulness-based cognitive therapy; MCI, mild cognitive impairment; MF, mindfulness; MiCBT, mindfulness-integrated cognitive behaviour therapy; MS, multiple sclerosis; ND, neurological disorder; NL, Netherlands; NR, Nt reported; PD, Parkinson's disease; PHQ, patient health questionnaire; PP, positive psychology; PST, problem-solving therapy; PsychoEd, Psychoeducation; QIDS, quick inventory of depressive symptomatology; s.d., standard deviation; SAD-Q, stroke aphasic depression questionnaire; SAS, self-rating anxiety; SDS,the self-rating depression scale; SE, Sweden; SEFT, supportive emotion focused training; SIGH-A, hamilton anxiety rating scale; SS, social support; TAU, treatment as usual; TBI, traumatic brain injury; UC, usual care; UK, Uniked Kingdom; UP, unified protocol; US, United States of America; WLC, waiting list control; yrs, years.

a For the moderator analyses relating to ND subgroups, the MS data were extracted separately from this study given there were sufficient sample sizes in these subgroups (i.e. 20 in each arm) to be included within the analyses. There were insufficient samples within the other subgroups and thus this data was not separately extracted nor included within the moderator analyses.

b Study reported multiple eligible treatment arms.

Studies were published between 2000 and 2023 with 23 trials (43%) published within the past 5 years. All studies examined depression outcomes with three reporting on two eligible intervention arms, thus data were extracted from 57 intervention and 54 control arms for depression. Twenty-nine (54%) studies also examined anxiety outcomes, and data was extracted from 29 intervention and 29 control arms.

Thirty-four (60%) interventions were delivered face-to-face, 13 (23%) digitally, seven (12%) through teletherapy, and three (5%) used multiple delivery modes. Most studies used inactive control groups (*k* = 41; 76%), including 23 (43%) treatment-as-usual or usual care groups and 18 (33%) waitlist-control groups. Twelve (22%) studies used an active control group, predominantly education (*k* = 7; 13%); one did not report control type. Thirty-one (57%) studies utilized a minimum baseline symptom severity inclusion criterion (i.e. participants met diagnostic criteria for a depressive or anxiety disorder or indicated clinical levels of symptoms).

### Meta-analytic results

Table 2 displays the results of the random-effects meta-analysis, heterogeneity (*I*^2^), between-study variance (*t*^2^), and publication bias (Egger's test) for depression and anxiety. Forest plots for depression and anxiety are available in online Supplementary Figs S1 and S2.

**Table 2.** Results of random effects meta-analyses and mixed effects moderator analyses

	Depression					Anxiety				
	*k*	Hedges' *g* [95% CI]	*I*^2^ [95% CI]	*t*^2^	*p* value	*k*	Hedges' *g* [95% CI]	*I*^2^ [95% CI]	*t*^2^	*p* value
All arms	57	0.45 [0.35–0.54]	64.60 [52.85–73.13]	0.08	<0.001*	29	0.38 [0.29–0.48]	27.70 [0.0–54.32]	0.02	<0.001*
Neurological disorder type					0.021*					0.043*
__Multiple sclerosis	14^a^	0.57 [0.34–0.81]	78.16 [63.84–86.81]	0.15	<0.001*	7^a^	0.41 [0.21–0.61]	37.62 [0.0–73.74]	0.03	<0.001*
__Stroke	10	0.28 [0.15–0.42]	0.0 [0.0–36.80]	0.0	<0.001*	–^b^				
__Epilepsy	9	0.34 [0.20–0.48]	0.0 [0.0–63.68]	0.0	<0.001*	5	0.20 [0.02–0.37]	0.0 [0.0–0.0]	0.00	0.025*
__Parkinson's disease	7	0.88 [0.51–1.24]	73.59 [43.41–87.68]	0.18	<0.001*	6	0.64 [0.38–0.90]	44.28 [0.0–77.95]	0.05	<0.001*
__Traumatic brain injury	7	0.31 [0.16–0.45]	0.0 [0.0–65.77]	0.0	<0.001*	4	0.20 [−0.09 to 0.49]	0.0 [0.0–65.34]	0.0	0.184
__Migraine	4	0.50 [0.03–0.97]	81.62 [52.26–92.93]	0.18	0.038*	4	0.42 [0.22–0.63]	8.31 [0.08–8.16]	0.0	<0.001*
Tailored CBT					0.904					0.739
__Yes	48	0.44 [0.34–0.55]	67.67 [56.42–76.02]	0.09	<0.001*	26	0.38 [0.28–0.48]	26.61 [0.0–54.75]	0.02	<0.001*
__No	9	0.43 [0.25–0.61]	32.92 [0.0–69.07]	0.03	<0.001*	3	0.46 [0.00–0.91]	56.65 [0.0–87.64]	0.09	0.049*
Delivery					0.478					0.458
__Face-to-face	34	0.47 [0.36–0.59]	54.55 [33.03–69.15]	0.07	<0.001*	19	0.40 [0.29–0.52]	13.42 [0.0–49.08]	0.01	<0.001*
__Digital	13	0.53 [0.33–0.73]	71.91 [50.84–83.95]	0.09	<0.001*	8	0.33 [0.17–0.49]	35.73 [0.07–1.58]	0.02	<0.001*
__Teletherapy	7	0.31 [0.01–0.61]	75.43 [48.01–88.38]	0.11	0.041*	–	–	–	–	–
Symptom severity inclusion					0.012*					0.019*
__Yes	34	0.53 [0.38–0.68]	73.70 [63.18–81.21]	0.14	<0.001*	14	0.51 [0.35–0.67]	42.30 [0.0–50.05]	0.04	<0.001*
__No	23	0.32 [0.24–0.40]	0.77 [0.0–45.82]	0.0	<0.001*	15	0.28 [0.18–0.39]	0.0 [0.0–75.79]	0.0	<0.001*
Outcome measure					0.014*					<0.001*
__HAM-D/HAM-A	11	0.61 [0.32–0.92]	79.78 [64.57–88.46]	0.20	<0.001*	4	0.81 [0.57–1.05]	0.0 [0.0–83.15]	0.0	<0.001*
__HADS-D/HADS-A	9	0.40 [0.24–0.56]	0.0 [0.0–26.62]	0.0	<0.001*	12	0.27 [0.14–0.40]	0.0 [0.0–50.05]	0.0	<0.001*
__PHQ-9/GAD-7	9	0.29 [0.17–0.41]	9.49 [0.0–68.37]	0.0	<0.001*	5	0.26 [0.10–0.41]	0.0 [0.0–57.97]	0.0	0.001*
__DASS-D/DASS-A	6	0.57 [0.18–0.95]	69.01 [27.00–86.84]	0.15	0.004*	4	0.52 [0.23–0.79]	32.46 [0.0–75.97]	0.03	<0.001*
__BDI	9	0.70 [0.38–1.02]	81.89 [66.81–90.12]	0.20	<0.001*	–	–	–	–	–
__CES-D	6	0.18 [0.02–0.34]	0.0 [0.0–0.63]	0.0	0.026*	–	–	–	–	–
__GDS	3	0.19 [−0.03 to 0.41]	0.0 [0.0–91.38]	0.0	0.098	–	–	–	–	–
Control type					0.048*					0.166
__Active	12	0.30 [0.15–0.44]	43.83 [0.0–71.39]	0.03	<0.001*	4	0.54 [0.31–0.75]	0.00 [0.0–57.16]	0.0	<0.001*
__Inactive	44	0.48 [0.37–0.60]	66.54 [54.15–75.58]	0.10	<0.001*	24	0.37 [0.26–0.48]	34.09 [0.0–59.88]	0.02	<0.001*
Risk of bias					0.488					0.360
__Low	11	0.53 [0.31–0.75]	71.54 [47.51–84.57]	0.10	<0.001*	4	0.31 [0.14–0.48]	1.51 [0.0–87.28]	0.00	<0.001*
__Some concerns	21	0.46 [0.28–0.64]	71.88 [56.45–81.85]	0.10	<0.001*	12	0.47 [0.30–0.64]	37.18 [0.0–68.25]	0.03	<0.001*
__High	25	0.39 [0.26–0.51]	48.45 [17.94–67.61]	0.05	<0.001*	13	0.34 [0.20–0.48]	20.97 [0.0–58.55]	0.01	<0.001*
		*b* [95% CI]	*I*^2^ [95% CI]	*t*^2^	*p* value		*b* [95% CI]	*I*^2^ [95% CI]	*t*^2^	*p* value
Sample size	57	−0.00 [−0.00 to 0.00]	63.76 [51.81–72.75]	0.08	0.300	29	−0.00 [−0.00 to 0.00]	24.16 [0.0–51.52]	0.02	0.162
Study year	57	0.01 [−0.02 to 0.03]	64.53 [52.91–73.28]	0.08	0.501	29	0.00 [−0.03 to 0.03]	30.21 [0.0–56.16]	0.02	0.979

a The multiple sclerosis subgroup data from Gandy et al. (2023) mixed RCT was included within the moderator analyses.

b Not enough studies to include in analyses.

*Significant at *p* < 0.05.

The overall pooled effect size for depression was small-to-moderate favoring the intervention (*g* = 0.45, 95% CI 0.35–0.54, *p* < 0.001), with a substantial, significant level of heterogeneity (*I*^2^ = 64.60, *p* < 0.001). Seven interventions were identified as potential outliers due to large effects (*gs* = 1.10–1.56) however the ‘one study removed procedure’ did not indicate any to have an influence on the pooled effect size (see online Supplementary Fig. S3).

The overall pooled effect size for anxiety was small, favoring the intervention (*g* = 0.38, 95% CI 0.29–0.48, *p* < 0.001), with a low, nonsignificant level of heterogeneity (*I*^2^ = 27.70, *p* = 0.09). No studies were identified as potential outliers.

### Moderator analyses

Table 2 displays results of subgroup analyses and meta-regression. Significant intervention effects for depression were observed for all ND types and moderator analysis revealed treatment effects significantly varied by ND type. The largest effects were observed in PD (*g* = 0.88, 95% CI 0.51–1.24), MS (*g* = 0.57, 95% CI 0.34–0.81), and migraine (*g* = 0.50, 95% CI 0.03–0.97), and smallest effects for epilepsy (*g* = 0.34, 95% CI 0.20–0.48), TBI (*g* = 0.31, 95% CI 0.16–0.45), and stroke (*g* = 0.28, 95% CI 0.15–0.42). Anxiety treatment effects also significantly differed by NDs, with moderate effects for PD (*g* = 0.64, 95% CI 0.38–0.90) and small effects for MS (*g* = 0.41, 95% CI 0.21–0.61), migraine (*g* = 0.42, 95% CI 0.22–0.63), and epilepsy (*g* = 0.20, 95% CI 0.02–0.37). Notably, the TBI group had nonsignificant effects (*g* = 0.20, 95% CI −0.09 to 0.49). See online Supplementary Figs S4 and S5 for forest plots distinguished by disorder type.

Effects on depression varied significantly by outcome measure, with moderate effects in studies using the BDI (*g* = 0.70, 95% CI 0.38–1.02), HAM-D (*g* = 0.61, 95% CI 0.32–0.92), and DASS-D (*g* = 0.57, 95% CI 0.18–0.95), and small effects with the HADS-D (*g* = 0.40, 95% CI 0.24–0.56), PHQ-9 (*g* = 0.29, 95% CI 0.17–0.41), and CES-D (*g* = 0.18, 95% CI 0.02–0.34). Three studies using the GDS to measure depression demonstrated nonsignificant treatment effects compared to controls (*g* = 0.19, 95% CI −0.03 to 0.41). Effects on anxiety also varied: large for the HAM-A (*g* *=* 0.81, 95% CI 0.57–1.05), moderate for the DASS-A (*g* = 0.52, 95% CI 0.23–0.79), and small for the HADS-A (*g* = 0.27, 95% CI 0.14–0.40) and GAD-7 (*g* = 0.26, 95% CI 0.10–0.41), though all subgroups had significant treatment effects relative to controls. Studies with and without a minimal symptom severity inclusion criterion were both efficacious compared to controls. However, those requiring a diagnosis or symptoms above a clinical cut-off at baseline reported greater intervention effects, specifically moderate effects for both depression (*g* = 0.54, 95% CI 0.39–0.70) and anxiety (*g* = 0.51, 95% CI 0.35–0.67), compared to small effects observed in those without a baseline symptom criterion (*g* = 0.32, 95% CI 0.24–0.40; *g* = 0.28, 95% CI 0.18–0.39 respectively).

Control group moderated treatment effects for depression, but not anxiety, outcomes. While both subgroups demonstrated significant treatment effects, larger effects were observed for depression trials using inactive (*g* = 0.48, 95% CI 0.37–0.60) than active controls (*g* = 0.30, 95% CI 0.15–0.44).

The effects of delivery mode, intervention tailoring, and risk of bias, sample size, and year of publication were nonsignificant.

### Study bias

The funnel plot for depression interventions was slightly asymmetric (see online Supplementary Fig. S6) with some larger studies falling past the right boundary of the funnel indicating particularly large effect sizes, corroborated by a significant Egger's test (*p* = 0.03). However, the trim and fill analysis suggested no missing studies and hence no revised effect size. The funnel plot for anxiety interventions appeared generally symmetric (see online Supplementary Fig. S7) and Egger's test was nonsignificant (*p* = 0.09), thus there was no indication of bias.

### Risk of bias

**Figure 2.**
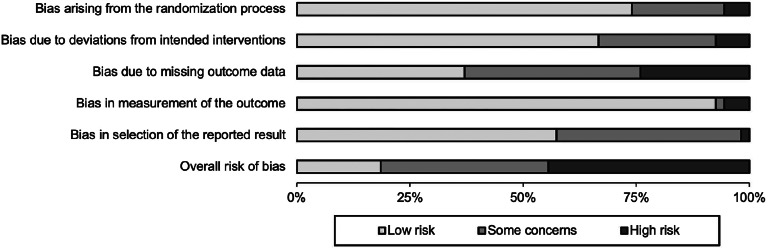
Summary of risk of bias.

Figure 2 shows a summary of the risk of bias ratings across the five domains. Ratings for each study are available in online Supplementary Fig. S8. Most studies (*k* = 24; 44%) had an overall high risk of bias, 20 (37%) indicated some concern, and 10 (19%) were low risk.

## Discussion

This systematic review and meta-analysis explored the effects of cognitive and behavioral interventions on depression and anxiety symptoms for people with a range of NDs. Across 54 RCTs (*n* = 5372), we observed small-to-moderate positive effects on depression across the 57 treatment arms, and small positive effects on anxiety across 29 treatment arms. These findings support the efficacy of cognitive and behavioral interventions for addressing common psychosocial challenges of NDs, which has been underscored as a key target of IGAP (World Health Organisation, 2023).

Our observed small-to-moderate effects provide important benchmarks for the existing literature. The magnitude of these effects are consistent with several smaller disorder-specific meta-analyses in PD (Ghielen et al., 2019), MS (Hind et al., 2014), and epilepsy (Li et al., 2023), as well as in adults with long-term physical health conditions (Scott et al., 2023). Contrary to previous meta-analyses (Barua et al., 2024), anxiety in people with TBI did not improve compared to controls, possibly due to our exclusion of trials with fewer than 20 participants per group. Our effect size for depression was similar to that identified in a meta-analysis of antidepressants for NDs (Price et al., 2011), which mirrors the short-term clinical equivalence of psychological and pharmacological treatments observed in the general population (Cuijpers et al., 2023). Nevertheless, our benchmarks of observed clinical effects are more modest than the moderate effects observed for psychological interventions for people within the general or ‘healthy’ population (Cuijpers et al., 2023).

Of interest, intervention effects were significantly smaller for trials which did not employ a minimum symptom severity inclusion criterion compared to those which did (e.g. depression *g* = 0.32 *v. g* = 0.54). The moderate effect sizes of those studies which used a severity criterion were more comparable to effect sizes observed in the general population (Cuijpers et al., 2023). These differences in effect sizes are somewhat unsurprising given participants with clinically meaningful baseline symptoms have more ‘room’ for improvement and associated inclusion criterion can ensure outcome relevance. Thus, interventions with broader inclusion criteria would benefit from analyses that account for baseline symptom severity when analyzing intervention effects (Gandy et al., 2023).

Importantly, significant intervention effects for depression compared to controls were found for all ND subgroups. However, there was evidence of differential efficacy across NDs for, with the largest overall depression effects observed in PD (*g* = 0.88, *k* = 7), moderate effects for MS (*g* = 0.57, *k* = 14) and migraine (*g* = 0.50, *k* = 4), whereas those with epilepsy (*g* = 0.34, *k* = 9) and TBI (*g* = 0.31, *k* = 7) and stroke (*g* = 0.28, *k* = 10) had small effects. For anxiety, significant intervention effects were observed for all available ND groups except TBI. This also resulted in evidence of differential efficacy across NDs, with moderate effects observed for PD (*g* = 0.64, *k* = 6), small effects for MS (*g* = 0.41, *k* = 7), migraine (*g* = 0.42, *k* = 4) and epilepsy (*g* = 0.20, *k* = 5), and non-significant effects for TBI (*g* = 0.20, *k* = 4). Due to insufficient trial numbers, we were unable to calculate anxiety outcomes for stroke. Nor were we able to include other disorders, such as dementia (*k* = 1) and mild cognitive impairment (*k* = 2) within subgroup analyses. We found no RCTs that met full criteria for patients with a FND or Huntington's disease, highlighting the need for future trials in these populations.

Our overall effect for depression in PD was larger than previous meta-analysis, which found a small effect (*g* = 0.37) (Ghielen et al., 2019). Interestingly, 3/7 (43%) of the included PD trials were conducted by the same team, and reported large effects (*gs* = 1.10–1.56) (Dobkin et al., 2011, 2020, 2021). These studies were not included in previous reviews and may have contributed to this observed trend. Our findings are consistent with moderate effects previously identified for MS (Hind et al., 2014) and offer a novel meta-analytic benchmark for depression effects in migraine. The smaller effects for epilepsy, stroke and TBI are also consistent with previous disorder-specific reviews in these conditions (Barua, Ahrens, Mehta, & Shao, 2021; Little, Byrne, & Coetzer, 2021). However, without direct comparisons of NDs within clinical trials we can only speculate as to these observed differences. In fact, only one study utilized a mixed ND sample and although it was underpowered to directly compare across disorders, participants with PD were the only subgroup which did not significantly improve (Gandy et al., 2023). Thus, it is unclear what may be underlying differences in results and there is a need for further research in this area.

Our results suggest that cognitive and behavioral interventions have smaller effects on reducing symptoms of anxiety than depression. It is possible that the interventions themselves were more oriented toward alleviating symptoms of depression than anxiety. No studies focused solely on anxiety compared to 26 (48%) studies which measured only depression. Of those which measured both outcomes, several reported anxiety as a secondary outcome. Future development of interventions which emphasize anxiety is warranted, particularly as anxiety is prevalent and can occur without depression in NDs (Munger Clary, Snively, & Hamberger, 2018).

We found intervention efficacy was not significantly moderated by delivery mode, corroborating meta-analyses in the general population (Kriston, Liebherz, & Köhnen, 2022; Zhang et al., 2022), and expanding these findings by also including teletherapy. These results are promising considering the upsurge in teletherapy since the COVID-19 pandemic (Meininger et al., 2022) and general paradigm shift toward digital mental health interventions (Andersson, Titov, Dear, Rozental, & Carlbring, 2019). These modalities provide options for accessing psychological care remotely which may be preferable to some people with NDs, particularly those with mobility restrictions and cognitive difficulties (Gandy et al., 2018).

Most studies used some form of intervention tailoring to suit people with NDs, which is recommended as best practice (Karekla et al., 2019; Sanders, Coppin, Moulson, Meola, & Meyrick, 2020). Specifically, 48 (84%) depression interventions and 27 (93%) anxiety interventions tailored their intervention by either providing ND-specific information or addressing common cognitive concerns. However, we found no evidence that this broad level tailoring resulted in larger intervention effects. It is possible that specific types of tailoring are important, including for outcomes beyond depression and anxiety. For instance, psychological interventions have been recommended in conjunction with cognitive remediation strategies to address functional difficulties that people with NDs experience (Gallagher et al., 2019), with evidence these combined treatments can improve quality of life, participation, and functioning in people with an ABI (Davies et al., 2023).

We found treatment effects for depression and anxiety significantly differed depending on the outcome measure used. Moderate-to-large effects were observed for studies using the Hamilton scales and BDI, whereas the PHQ-9 and CES-D had small effects. These findings are largely consistent with previous meta-analysis of cognitive behavioral interventions for chronic health conditions (Scott et al., 2023). Of note, the Hamilton rating scales are investigator-administered measures, while the others are self-report. It is possible that investigator-administration introduces bias in participant reporting that inflates effects. Alternatively, investigators may be better equipped to detect symptom improvement. Indeed, concerns have been raised about the validity of generic self-report measures in NDs due to common somatic-symptom items (e.g. dizziness), which can be confounded by neurological symptoms (Cella et al., 2012; Scott et al., 2019). Future trials need to ensure outcome measures are recommended for ND populations. Notably, the GDS showed non-significant effects in studies with dementia and MCI samples.

Most studies utilized an inactive control group (*k* = 44; 76%). The remaining 12 studies used a form of active control, typically forms of education. Subgroup analyses found moderate overall effects for depression with inactive controls (*g* = 0.48) compared to small effects with active controls (*g* = 0.30). These trends are consistent with meta-analysis in the general population and other chronic health conditions (e.g. Scott et al., 2023) but were not observed for anxiety in our study. The use of active controls in clinical trials are known to produce smaller effects given they attempt to control for non-specific effects of interventions. However, given the relatively small number of trials to utilize an active control (only *k* = 4 for anxiety) some caution is needed in interpreting the current results.

### Limitations

A limitation of studies was the higher overall dropout rate (pre- to post-intervention) across participants receiving interventions (17% dropout) compared to controls (11% dropout). Seventeen (30%) interventions reported a dropout rate ≥20%, a level considered a concern for attrition bias (Schulz & Grimes, 2002), compared to only seven (13%) in control groups. Given these dropout rates, it is important that studies conduct intent-to-treat analysis that manages missing data appropriately (e.g. by examining patterns of missingness and/or replacing missing data). However, this was not consistently done or clearly reported, contributing to many studies (43%) having a high RoB. Although RoB did not moderate intervention effects, future research could improve on trial quality and some caution is needed when interpreting the current results.

While our study included many studies (*k* = 54), subgroup analyses may still be underpowered to draw firm conclusions. Furthermore, there was considerable variability in the follow-up periods, which precluded an assessment of longevity of intervention effects. Future research would benefit from conducting further trials for managing anxiety in NDs, utilizing larger samples, and using active control methods. Despite contacting authors, we were unable to include nine trials due to missing data. Thus, it is recommended that any future trials clearly report this information, including both pre- and post-intervention means and s.d. scores, as well as the number of participants included within analyses for each study arm. Finally, across 14 studies to report race, most reported a high proportion of White/Caucasian participants (~86%), which raises concerns about the diversity of samples. Thus, we further recommend that future studies report key sociodemographic sample characteristics to contextualize the results and generalizability of findings. Increased efforts to recruit representative samples may also need further consideration.

## Conclusions

Our findings suggest that cognitive and behavioral interventions are an efficacious treatment option for symptoms of depression and anxiety in adults with a range of common NDs, which may be of interest to researchers, clinicians, and policymakers involved in their care and management of NDs. This includes informing referral decisions of neurologists and neurology care settings who treat many patients with depression and anxiety but rarely have capacity to manage these comorbidities. Future research into the role of cognitive and behavioral interventions within routine neurological care settings is a crucial next step.

## Supporting information

Gandy et al. supplementary material Gandy et al. supplementary material

## Supplementary material

The supplementary material for this article can be found at https://doi.org/10.1017/S0033291724001995.
