# Supplementary material for: Cognitive behavioral interventions for depression and anxiety in adults with neurological disorders: a systematic review and meta-analysis
Source: Psychol Med. 2024 Sep 25;54(12):3237–50. doi: 10.1017/S0033291724001995 (PMC11496241; doi:10.1017/S0033291724001995)
Supplement: Gandy et al. supplementary material [file S0033291724001995sup001.docx]

**Supplemental Online Content**

Cognitive and Behavioral Interventions for Depression and Anxiety in Adults with Neurological Disorders: A Systematic Review and Meta-analysis

**Methods S 1.** Electronic Search Strategy

**Table S 1.** Additional Study Characteristics

**Figure S 1.** Forest Plot of Effects of Interventions for Depression

**Figure S 2.** Forest Plot of Effects of Interventions for Anxiety

**Figure S 3.** Forest Plot of Effects with One Study Removed for Interventions on Depression

**Figure S 4.** Forest Plot of Effects of Interventions for Depression by Neurological Disorder Type

**Figure S 5.** Forest Plot of Effects of Interventions for Anxiety by Neurological Disorder Type

**Figure S 6.** Funnel Plot for Interventions on Depression

**Figure S 7.** Funnel Plot for Interventions on Anxiety

**Figure S 8.** Results of the Cochrane Risk-of Bias Tool for Randomised Controlled Trials (RoB-2)

**References. S 1.** References of Included Studies

**Supplementary Methods 1.** Electronic Search Strategy

| **EMBASE**    1 exp neurologic disease/  2 (neurologic* or epilep* or sclerosis or parkinson* or huntington* or stroke* or cerebral palsy or brain injur* or migraine or dementia* or motor neuron* or spinal cord injur*).ti,ab,kw.  3 1 or 2  4 exp depression/  5 exp anxiety/  6 (depress* or anxi* or mood or affect* or phobi* or panic*).ti,ab,kw.  7 4 or 5 or 6  8 exp cognitive behavioral therapy/  9 (cognitive behavio* or (mindfulness adj2 cognitive) or "acceptance and commitment" or problem solv* or dialectical behavio* or behavio* therap* or psychotherap* or cognitive therap* or counsell* or CBT or ACT or MBCT or PST).ti,ab,kw.  10 8 or 9  11 3 and 7 and 10  12 (randomi* controlled trial* or controlled clinical trial* or random* or trial*).ti,ab,kw.  13 11 and 12 |
| --- |
| **PSYCINFO**    1 exp nervous system disorders/  2 (neurologic* or epilep* or sclerosis or parkinson* or huntington* or stroke* or cerebral palsy or brain injur* or migraine or dementia* or motor neuron* or spinal cord injur*).mp.  3 1 or 2  4 exp Late Life Depression/ or exp Recurrent Depression/ or exp "Depression (Emotion)"/ or exp "Long-term Depression (Neuronal)"/ or exp Postpartum Depression/ or exp Cortical Spreading Depression/ or exp Treatment Resistant Depression/ or exp Major Depression/ or exp Beck Depression Inventory/ or exp Depression Screening/ or exp Atypical Depression/ or exp Reactive Depression/ or exp Endogenous Depression/  5 exp anxiety/  6 (depress* or anxi* or mood or affect* or phobi* or panic*).mp.  7 4 or 5 or 6  8 exp cognitive behavior therapy/  9 (cognitive behavio* or (mindfulness adj2 cognitive) or "acceptance and commitment" or problem solv* or dialectical behavio* or behavio* therap* or psychotherap* or cognitive therap* or counsell* or CBT or ACT or MBCT or PST).mp.  10 8 or 9  11 3 and 7 and 10  12 (randomi* controlled trial* or controlled clinical trial* or random* or trial*).mp.  13 11 and 12 |
| **CENTRAL**  #1 MeSH descriptor: [Nervous System Diseases] explode all trees  #2 (neurologic*):ti,ab,kw OR (epilep*):ti,ab,kw OR (sclerosis):ti,ab,kw OR (parkinson*):ti,ab,kw OR (huntington*):ti,ab,kw OR (stroke*):ti,ab,kw OR (cerebral palsy):ti,ab,kw OR (spinal cord injur*):ti,ab,kw OR (brain injur*):ti,ab,kw OR (migraine):ti,ab,kw OR (dementia):ti,ab,kw OR (motor neuron*):ti,ab,kw  #3 #1 or #2  #4 MeSH descriptor: [Depression] explode all trees  #5 MeSH descriptor: [Anxiety] explode all trees  #6 (depress*):ti,ab,kw OR (anxi*):ti,ab,kw OR (mood):ti,ab,kw OR (affect*):ti,ab,kw OR (phobi*):ti,ab,kw OR (panic):ti,ab,kw  #7 #4 or #5 or #6  #8 MeSH descriptor: [Cognitive Behavioral Therapy] explode all trees  #9 (cognitive behav*):ti,ab,kw OR ("mindfulness-based cognitive"):ti,ab,kw OR ("acceptance and commitment"):ti,ab,kw OR (problem solv*):ti,ab,kw OR (dialectical behav*):ti,ab,kw OR (behav* therapy):ti,ab,kw OR (psychotherap*):ti,ab,kw OR (cognitive therap*):ti,ab,kw OR (counsell*):ti,ab,kw OR (mindfulness adj2 cognitive):ti,ab,kw OR (CBT):ti,ab,kw OR (ACT):ti,ab,kw OR (MBCT):ti,ab,kw OR (PST):ti,ab,kw  #10 #8 or #9  #11 #3 and #7 and #10  #12 (randomi* controlled trial*):ti,ab,kw  #13 (controlled clinical trial*):ti,ab,kw  #14 (trial*):ti,ab,kw  #15 (random*):ti,ab,kw  #16 #12 or #13 or #14 or #15  #17 #11 and #16 |
| **MEDLINE**    1 exp Nervous System Diseases/  2 (neurologic* or epilep* or sclerosis or parkinson* or huntington* or stroke* or cerebral palsy or brain injur* or migraine or dementia* or motor neuron* or spinal cord injur*).ti,ab,kw.  3 1 or 2  4 exp Depression/  5 exp anxiety/  6 (depress* or anxi* or mood or affect* or phobi* or panic*).ti,ab,kw.  7 or/4-6  8 exp Cognitive Behavioral Therapy/  9 (cognitive behavio* or (mindfulness adj2 cognitive) or "acceptance and commitment" or problem solv* or dialectical behavio* or behavio* therap* or psychotherap* or cognitive therap* or counsell* or CBT or MBCT or ACT or PST).ti,ab,kw.  10 8 or 9  11 3 and 7 and 10  12 (randomi* controlled trial* or controlled clinical trial* or random* or trial*).ti,ab,kw.  13 11 and 12 |

**Supplementary Table 1.** Additional Study Characteristics

| **Study/ Country** | **Education**  M (SD) = Years of Education | **Race and/or Ethnicity** | **Sessions/ weeks of intervention** | **Primary outcome; Dep, Anx** |
| --- | --- | --- | --- | --- |
| A’Campo et al. (2010) / NL | Edu until 18 yrs (48.4%), Higher edu (51.6%) | NR | 8/8 | Y, N/A |
| Assonov, et al. (2021) / UK | M = 14, range = 12-16.75 yrs | NR | 6/6 | Y, Y |
| Bahrani, et al. (2017) / IR | Diploma (51.1%), Associate degree (10.6%), Bachelor (34.0%), Master (4.3%) | NR | 8/6 | Y, Y |
| Bailey, et al. (2017) / US | NR | NR | 12/6 | Y, N/A |
| Bedard, et al. (2014) / CA | Elementary (3.9%), Some sec (10.5%), Completed sec (25%), Some post-sec (12.8%), Completed post-sec (46.2%) | NR | 10/10 | Y, N/A |
| Bell, et al. (2017) / US | M = 13.4, SD = 1.81 yrs | Black or African American (8.15%), White (76.97%), Other (14.89%) / Hispanic or Latino (18.3%), Non-Hispanic or Latino (81.2%), Unknown (0.6%) | 12/24 | N, N/A |
| Belleville, et al. (2018) / CA | M = 14.75, SD = 3.63 yrs | NR | 8/8 | Y, Y |
| Boele, et al. (2018) / NL | Low (9.8%), Middle (43.9%), High (46.3%) | NR | 5/5 | Y, N/A |
| Boeschoten, et al. (2017) / NL | Low (1.2%), Middle (53.2%), High (45.6%) | NR | 5/Up to 10 | Y, N |
| Bogosian, et al. (2022) / UK | College or higher edu (93.3%) | White British (96.7%) | 8/8 | Y, Y |
| Bromberg, et al. (2012) / US | <11^th^ grade (0.6%), HS or GED (17.2%), 2 yrs of college / AA degree / technical school training (22.2%), College grad (33.4%), Master’s degree (20.0%), Doctoral / medical / law degree (6.1%) | White/non-Hispanic (87.5%), Black/African American (4.9%), Asian American (2.2%), Hispanic/Latino (2.2%), Native Hawaiian (0.5%), Other (2.7%) | Up to 9/4 | Y, Y |
| Ciechanowski, et al. (2010) / US | Edu beyond HS (56.3) | Racial/ethnic minority (27.5%) | 8/19 | Y, N/A |
| Dindo, et al. (2020) / US | >12 yrs edu (57.3%) | Caucasian race (76.7%) | 1/1 | Y, N |
| Dobkin, et al. (2021) / US | Some HS, but no diploma (3.3%), HS or GED (24.4%), Some college, no degree (47.8%), College grad or Grad/professional degree (24.4%) | White (92.2%), Black or African American (6.7%), American Indian (1.1%), Hispanic (4.4%) | Up to 16/26 | Y, N |
| Dobkin, et al. (2020) / US | HS diploma/some college (31.9%), College degree (36.11%), Grad degree (32.0%) | NR | Up to 16/26 | Y, N |
| Dobkin, et al. (2011) / US | HS diploma (15%), Some college (17%), College degree (24%), Grad degree (44%) | White (93%), Asian (5%), Black (1%), Pacific Islander (1%) | 10/10 | Y, N |
| Ehde, et al. (2015) / US | HS or less (9.2%), Tech or some college (25.8%), College degree or higher (65%) | Non-Hispanic white (83.4%), Non-Hispanic black (11.7%), Hispanic & >1 race (1.8%), Non-Hispanic & >1 race (3.1%) | 8/8 | Y, N/A |
| Exner, et al. (2021) / DE | M = 13.7, SD = 2.3 yrs | NR | M = 3.1/33.6 | N, N/A |
| Fann, et al. (2015) / US | GED or less (12%), HS diploma (10%), Tech/ Voc./ Some college (52%), College degree (26%) | Non-Hispanic white (90%), Other (10%) | 12/12 | Y, N/A |
| Feng, et al. (2022) / CN | M = 14.53, SD = 2.51 yrs | NR | 8/8 | Y, Y |
| Fischer, et al. (2015) / DE | 9 yrs (13.3%), 10 yrs (38.9%), 13 yrs (46.7%) | NR | 10/9 | Y, N/A |
| Fraser, et al. (2015) / US | M = 15.0, SD = 2.5 yrs | White (81%), Black (8%), Latino (11%), Asian (4%), Other (2%) | 8/8 | Y, Y |
| Gandy, et al. (2023) / AU | HS (20.9%), Trade certificate (13.0%), Under-grad/associate diploma (24.7), Higher research degree (41.4%) | NR | 6/10 | Y, Y |
| Gandy, et al. (2014) / AU | Sec school / vocational edu (51.1%), Tert edu (48.9%) | NR | 9/9 | Y, N |
| Gold, et al. (2023) / DE, US | NR | White (67.2%), African American or Black (1.9%), Hispanic or Latino/a (0.4%), Other (0.8%), Not provided (29.8%) | 11/12 | Y, N/A |
| Graziano, et al. (2014) / IT | 8 yrs (32.9%), 13 yrs (47.6%), >13 yrs (19.5) | NR | 5/26 | Y, N/A |
| Hum, et al. (2019) / CA | NR | Caucasian (75%), Asian (6.8%), African / Caribbean (6.8%), Middle Eastern (2.3%), Other / mixed (4.5%), Unknown / prefer not to answer (4.5%) | 8/8 | Y, N/A |
| Johnson, et al. (2020) / US | M = 14.6, SD = 2.3 yrs | White (89%), Black (6%), Latino (6%), Asian (4%), Native American (4%), Other (2%) | 8/8 | Y, Y |
| Kirkness, et al. (2017) / US | NR | Hispanic ethnicity (5%), >1 race (14%), White (79%), Black (5%), Asian (2%) | 6/8 | Y, N/A |
| Kraepelien, et al. (2020) / SE | College/university educated (28.6%) | NR | Up to 10/10 | N, N |
| Liu et al. (2023) / CN | Pri (3.6%), HS (64.0%), Junior college (19.4%), Bachelor (12.9%) | NR | 7/4 | Y, N/A |
| Lin, et al. (2023) / HK | Pri edu or below (54.4%), Sec edu or above (45.6%) | NR | 10/18 | Y, N/A |
| Lincoln, et al. (2011) / UK | NR | NR | 6/12 | N, N |
| Majumdar, et al. (2019) / UK | M age left edu = 17.8, SD = 3.0 years | NR | 4/4 | Y, N |
| Martin, et al. (2015) / AU | <Year 12 (9.1%), Year 12 (16.7%), Certificate/diploma (24.2%), Under-grad degree (30.3%), Post-grad degree (19.7%) | NR | 12/12 | Y, N |
| Meyer, et al. (2019) / DE | Did not finish school (8.5%), Basic-level HS (1.5%), Medium-level HS (12%), Higher-level HS (29.5%), Highest-level HS (15.5%), University degree (14%), Other edu qualification (19%) | NR | NR/Up to 26 | Y, N |
| Migliorini, et al. (2016) / AU | NR | NR | 10/10 | Y, Y |
| Mohr, et al. (2005) / US | M = 15.4, SD = 2.6 yrs | White (89.8%), African American (4.7%), Latin American (1.6%), Native American (1.6%), Asian or Pacific Islander (0.8%), Other (1.6%) | 16/16 | Y, N/A |
| Moonen, et al. (2021) / NL | M = 13.8, SD = 3.7 yrs | NR | 10/10 | N, Y |
| Nazari, et al. (2020) [a] / IR | Pri edu (17.2%), Bachelor (46.9%), Master+ (35.9%) | NR | 14/14 | Y, Y |
| Nazari, et al. (2020) [b] / IR | Pri edu (17.1%), Higher edu (82.9%) | NR | 12/12 | Y, Y |
| Niu, et al. (2022) / CN | Low (31.7%), Middle (41.3%), High (26.9%) | NR | 5/2 | Y, N/A |
| Pahlavanzadeh, et al. (2017) / IR | NR | NR | 8/8 | Y, Y |
| Ponsford, et al. (2016) / AU | M = 12.8, SD = 3.5 yrs | NR | 12/12 | Y, Y |
| Potter, et al. (2016) / UK | M = 15.0, SD = 3.0 yrs | NR | 12/12 | N, N |
| Sadeghi-Bahmani, et al. (2022) / IR | NR | NR | 8/8 | Y, N/A |
| Schröder, et al. (2014) / DE | HS (47.4%) | NR | 10/9 | Y, N/A |
| Simshauser, et al. (2021) / DE | NR | NR | 8/8 | N, N |
| Spruill, et al. (2021) / US | <HS (45.8%), HS grad (45.8%), college grad (8.3%), not working for pay (69.4%) | Born outside USA (72.1%), including Mexico (28.6%), Ecuador (22.4%), Dominican Republic (20.4%), Puerto Rico (18.4%), remaining from Colombia, El Salvador, Peru. | 8/8 | Y, N/A |
| Sun, et al. (2022) / CN | Illiterate (16.9%), Pri school (50.8%), Junior school (21.5%), HS+ (10.8%) | NR | 6/6 | Y, N/A |
| Thomas, et al. (2019) / UK | NR | White (97.9%), Asian (2.1%) | 15/17.3 | Y, N/A |
| Thomas, et al. (2012) / UK | NR | NR | 20/13 | Y, N/A |
| Visser, et al. (2016) / NL | High edu level (34.9%) | NR | 8/8 | N, N/A |
| Wang, et al. (2020) / CN | Illiterate (3.0%), Elementary (29.9%), Sec (38.1%), Technical sec (3.0%), Junior college (1.5%), Bachelor+ (24.6%) | Han (98.5%), Tibetan (1.5%) | 8/8 | Y, N/A |

*Note.* Edu, education; grad, graduate; HS, high school; M, mean; N/A, not applicable; NR, not reported; pri, primary; SD, standard deviation; sec, secondary; tert, tertiary; yrs, years.

**Supplementary Figure 1.** Forest Plot of Effects of Interventions on Depression


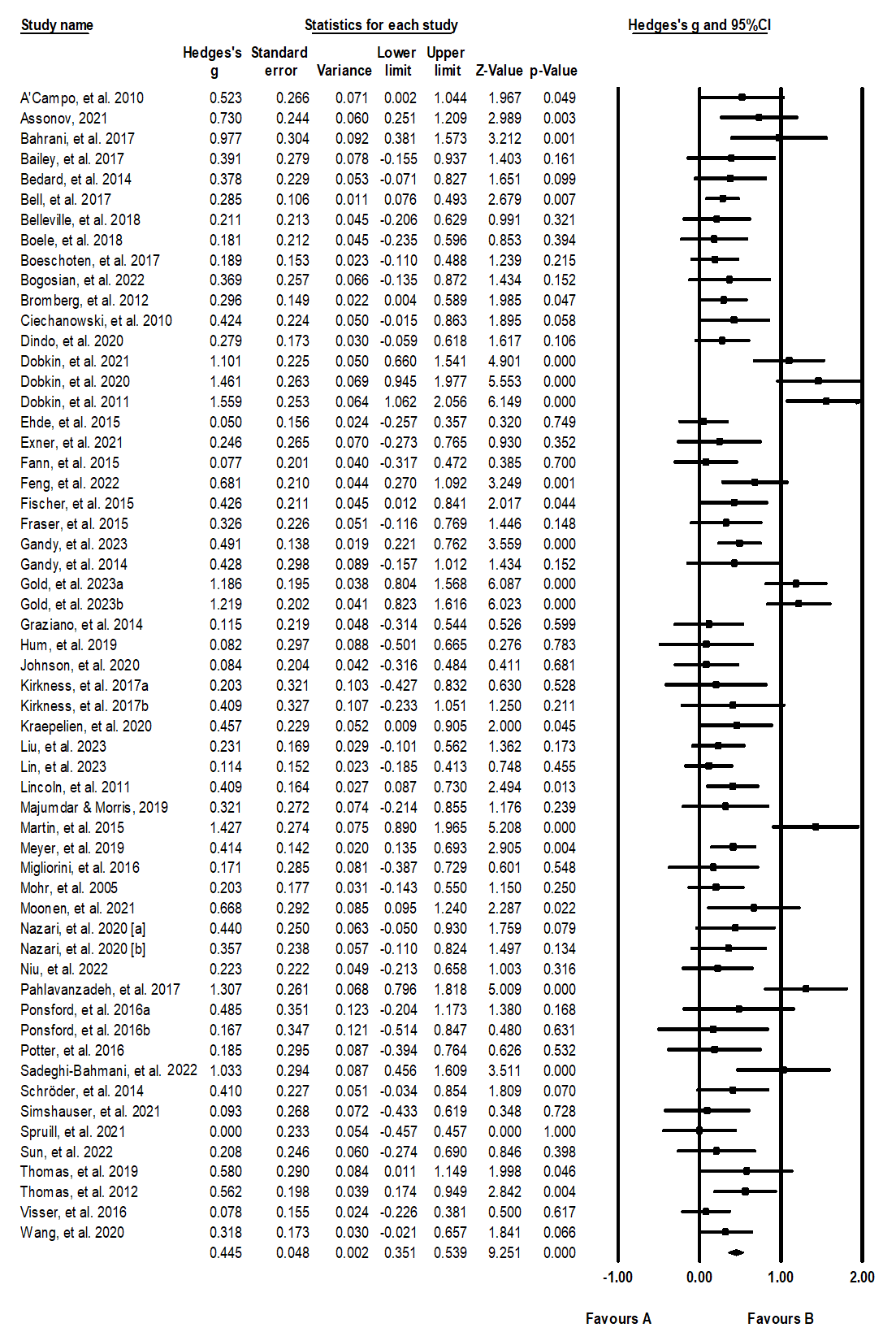


**Supplementary Figure 2.** Forest Plot of Effects of Interventions on Anxiety


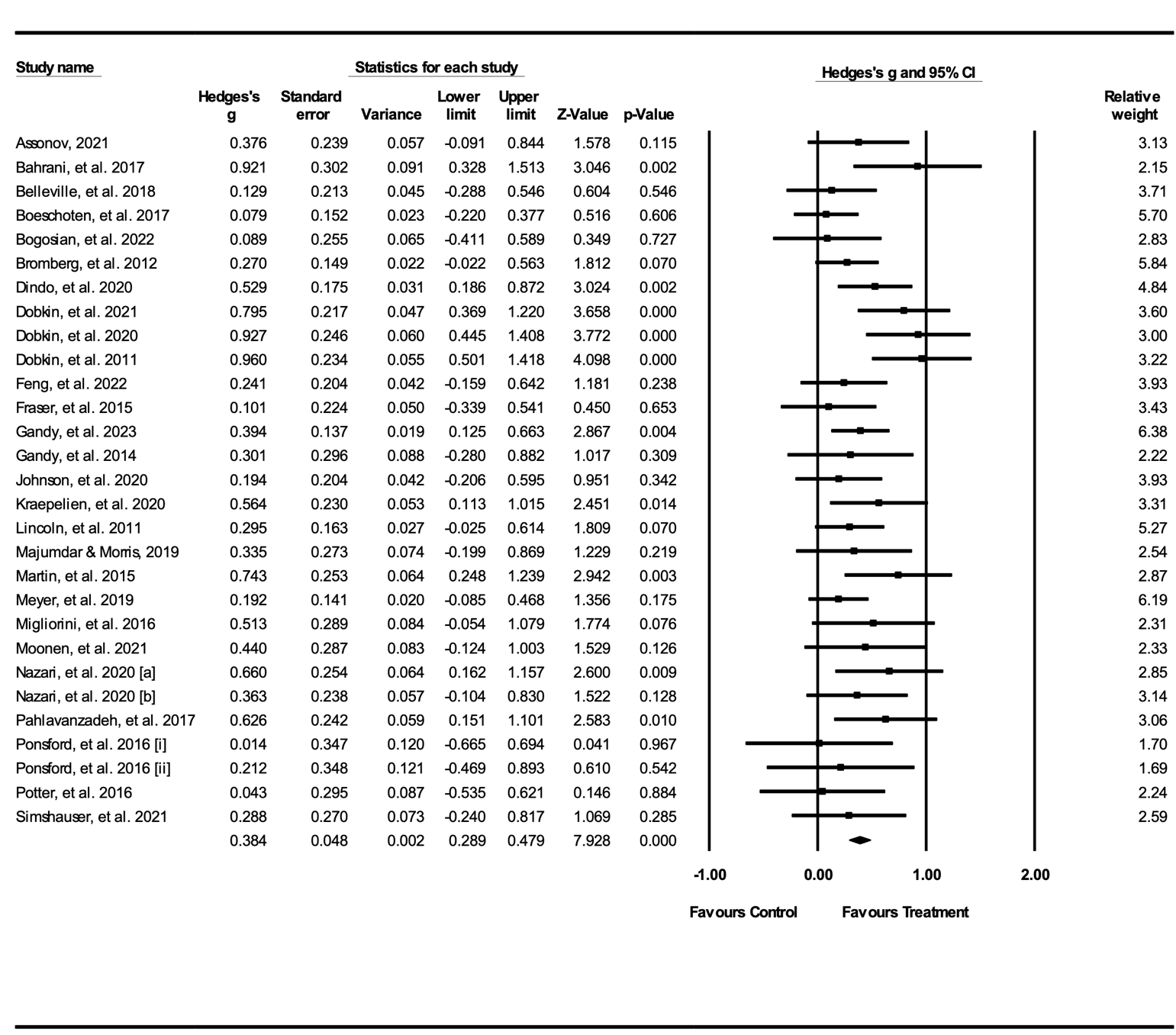


**Supplementary Figure 3.** Forest Plot of Effects with One Study Removed for Interventions on Depression

**Supplementary Figure 4.** Forest Plot of Effects of Interventions on Depression by Neurological Disorder Type

**Supplementary Figure 5.** Forest Plot of Effects of Interventions for Anxiety by Neurological Disorder Type

MS = Multiple Sclerosis, PD = Parkinson’s disease; TBI = Traumatic Brain Injury

**Supplementary Figure 6.** Funnel Plot for Interventions on Depression

 

**Supplementary Figure 7.** Funnel Plot for Interventions on Anxiety

 

**Supplementary Figure 8.** Results of the Cochrane Risk-of Bias Tool for Randomised Controlled Trials (RoB-2)

The RoB-2 examines five domains: randomization (D1), deviations from the intended intervention (e.g. changes to planned intervention; D2), handling of missing data (D3), outcome measurement (e.g. assessor blinding; D4), and reporting of results (e.g. adherence to analysis plan; D5).

Each domain was assessed using standard closed-ended questions provided in the tool, which dictate the level of risk for the domain. The overall level of risk for the study was determined based on domain ratings: studies with a domain rated as ‘high risk’ or multiple domains rated as having ‘some concerns’ and reduce confidence in the result were given an overall rating of ‘high risk’; studies with 1-2 domains rated as ‘some concerns’ were rated to have ‘some concerns’ overall; studies with all domains rated as ‘low risk’ were rated ‘low risk’ overall.

Items relating to concealment of treatment allocation to participants were omitted, given the inherent inability to blind participants to their treatment in psychotherapy trials.


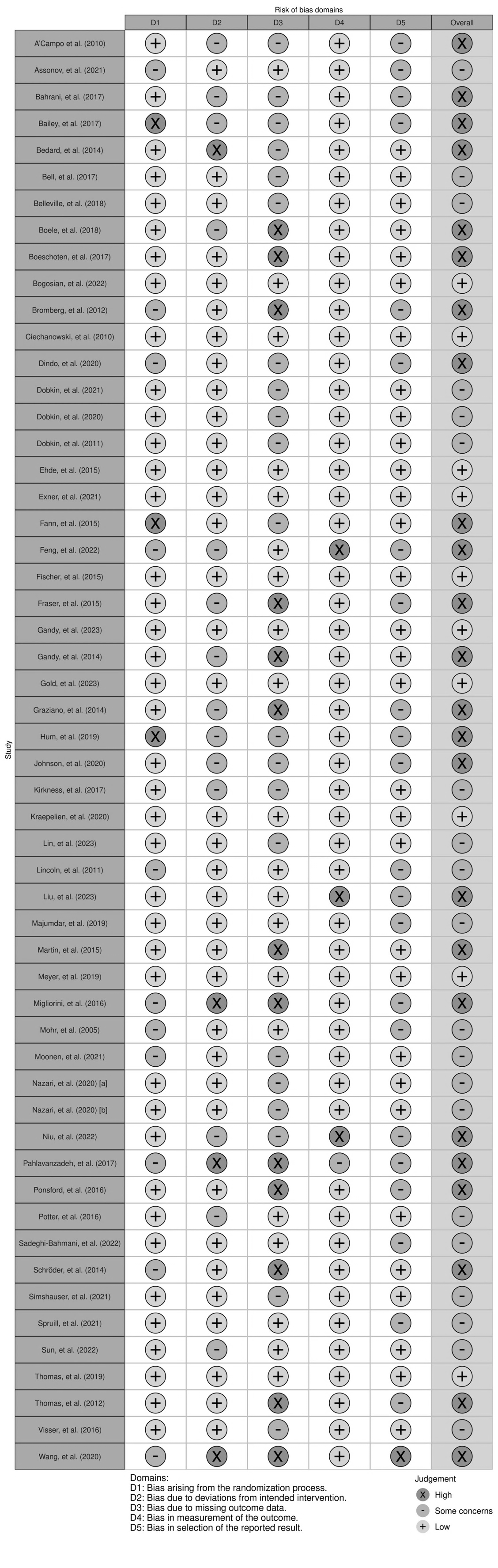


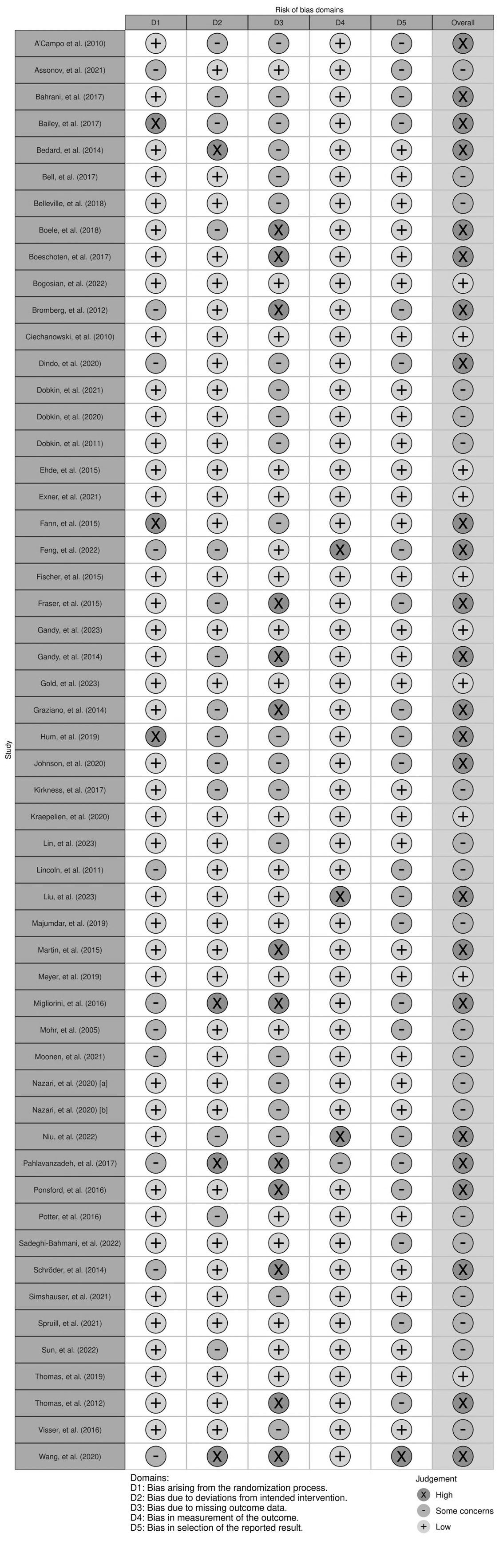


**Supplementary References**. References of Included Studies

A'Campo, L. E., Wekking, E. M., Spliethoff-Kamminga, N. G., Le Cessie, S., & Roos, R. A. (2010). The benefits of a standardized patient education program for patients with Parkinson's disease and their caregivers. *Parkinsonism Relat Disord, 16*(2), 89-95. doi:10.1016/j.parkreldis.2009.07.009

Assonov, D. (2021). Two-Step Resilience-Oriented Intervention for Veterans with Traumatic Brain Injury: A Pilot Randomized Controlled Trial. *Clin Neuropsychiatry, 18*(5), 247-259. doi:10.36131/cnfioritieditore20210503

Bahrani, S., Zargar, F., Yousefipour, G., & Akbari, H. (2017). The Effectiveness of Mindfulness-Integrated Cognitive Behavior Therapy on Depression, Anxiety, and Stress in Females with Multiple Sclerosis: A Single Blind Randomized Controlled Trial. *Iranian Red Crescent Medical Journal, In press*. doi:10.5812/ircmj.44566

Bailey, E. M., Stevens, A. B., LaRocca, M. A., & Scogin, F. (2017). A Randomized Controlled Trial of a Therapeutic Intervention for Nursing Home Residents With Dementia and Depressive Symptoms. *Journal of Applied Gerontology, 36*(7), 895-908. doi:10.1177/0733464815627956

Bédard, M., Felteau, M., Marshall, S., Cullen, N., Gibbons, C., Dubois, S., . . . Moustgaard, A. (2014). Mindfulness-based cognitive therapy reduces symptoms of depression in people with a traumatic brain injury: results from a randomized controlled trial. *J Head Trauma Rehabil, 29*(4), E13-22. doi:10.1097/HTR.0b013e3182a615a0

Bell, K. R., Fann, J. R., Brockway, J. A., Cole, W. R., Bush, N. E., Dikmen, S., . . . Temkin, N. (2017). Telephone Problem Solving for Service Members with Mild Traumatic Brain Injury: A Randomized, Clinical Trial. *Journal of Neurotrauma, 34*(2), 313-321. doi:10.1089/neu.2016.4444

Belleville, S., Hudon, C., Bier, N., Brodeur, C., Gilbert, B., Grenier, S., . . . Gauthier, S. (2018). MEMO+: Efficacy, Durability and Effect of Cognitive Training and Psychosocial Intervention in Individuals with Mild Cognitive Impairment. *J Am Geriatr Soc, 66*(4), 655-663. doi:10.1111/jgs.15192

Boele, F. W., Klein, M., Verdonck-de Leeuw, I. M., Cuijpers, P., Heimans, J. J., Snijders, T. J., . . . On behalf of the Dutch Society for, N.-O. (2018). Internet-based guided self-help for glioma patients with depressive symptoms: a randomized controlled trial. *Journal of Neuro-Oncology, 137*(1), 191-203. doi:10.1007/s11060-017-2712-5

Boeschoten, R. E., Dekker, J., Uitdehaag, B. M., Beekman, A. T., Hoogendoorn, A. W., Collette, E. H., . . . van Oppen, P. (2017). Internet-based treatment for depression in multiple sclerosis: A randomized controlled trial. *Multiple Sclerosis Journal, 23*(8), 1112-1122. doi:10.1177/1352458516671820

Bogosian, A., Hurt, C. S., Hindle, J. V., McCracken, L. M., Vasconcelos e Sa, D. A., Axell, S., . . . Cubi-Molla, P. (2022). Acceptability and Feasibility of a Mindfulness Intervention Delivered via Videoconferencing for People With Parkinson’s. *Journal of Geriatric Psychiatry and Neurology, 35*(1), 155-167. doi:10.1177/0891988720988901

Bromberg, J., Wood, M. E., Black, R. A., Surette, D. A., Zacharoff, K. L., & Chiauzzi, E. J. (2012). A Randomized Trial of a Web-Based Intervention to Improve Migraine Self-Management and Coping. *Headache: The Journal of Head and Face Pain, 52*(2), 244-261. doi:10.1111/j.1526-4610.2011.02031.x

Ciechanowski, P., Chaytor, N., Miller, J., Fraser, R., Russo, J., Unutzer, J., & Gilliam, F. (2010). PEARLS depression treatment for individuals with epilepsy: a randomized controlled trial. *Epilepsy Behav, 19*(3), 225-231. doi:10.1016/j.yebeh.2010.06.003

Dindo, L. N., Recober, A., Calarge, C. A., Zimmerman, B. M., Weinrib, A., Marchman, J. N., & Turvey, C. (2020). One-Day Acceptance and Commitment Therapy Compared to Support for Depressed Migraine Patients: a Randomized Clinical Trial. *Neurotherapeutics, 17*(2), 743-753. doi:10.1007/s13311-019-00818-0

Dobkin, R. D., Mann, S. L., Gara, M. A., Interian, A., Rodriguez, K. M., & Menza, M. (2020). Telephone-based cognitive behavioral therapy for depression in Parkinson disease. *Neurology, 94*(16), e1764. doi:10.1212/WNL.0000000000009292

Dobkin, R. D., Mann, S. L., Weintraub, D., Rodriguez, K. M., Miller, R. B., St. Hill, L., . . . Interian, A. (2021). Innovating Parkinson's Care: A Randomized Controlled Trial of Telemedicine Depression Treatment. *Movement Disorders, 36*(11), 2549-2558. doi:10.1002/mds.28548

Dobkin, R. D., Menza, M., Allen, L. A., Gara, M. A., Mark, M. H., Tiu, J., . . . Friedman, J. (2011). Cognitive-Behavioral Therapy for Depression in Parkinson's Disease: A Randomized, Controlled Trial. *American Journal of Psychiatry, 168*(10), 1066-1074. doi:10.1176/appi.ajp.2011.10111669

Ehde, D. M., Elzea, J. L., Verrall, A. M., Gibbons, L. E., Smith, A. E., & Amtmann, D. (2015). Efficacy of a Telephone-Delivered Self-Management Intervention for Persons With Multiple Sclerosis: A Randomized Controlled Trial With a One-Year Follow-Up. *Archives of Physical Medicine and Rehabilitation, 96*(11), 1945-1958.e1942. doi:10.1016/j.apmr.2015.07.015

Exner, C., Doering, B. K., Conrad, N., Künemund, A., Zwick, S., Kühl, K., . . . Rief, W. (2022). Integrated neuropsychological and cognitive behavioural therapy after acquired brain injury: A pragmatic randomized clinical trial. *Neuropsychological Rehabilitation, 32*(7), 1495-1529. doi:10.1080/09602011.2021.1908902

Fann, J. R., Bombardier, C. H., Vannoy, S., Dyer, J., Ludman, E., Dikmen, S., . . . Temkin, N. (2015). Telephone and in-person cognitive behavioral therapy for major depression after traumatic brain injury: a randomized controlled trial. *J Neurotrauma, 32*(1), 45-57. doi:10.1089/neu.2014.3423

Feng, H. X., Wang, M. X., Zhao, H. M., Hou, X. X., Xu, B., Gui, Q., . . . Xue, S. R. (2022). Effect of cognitive behavioral intervention on anxiety, depression, and quality of life in patients with epilepsy. *Am J Transl Res, 14*(7), 5077-5087.

Fischer, A., Schröder, J., Vettorazzi, E., Wolf, O. T., Pöttgen, J., Lau, S., . . . Gold, S. M. (2015). An online programme to reduce depression in patients with multiple sclerosis: a randomised controlled trial. *Lancet Psychiatry, 2*(3), 217-223. doi:10.1016/s2215-0366(14)00049-2

Fraser, R. T., Johnson, E. K., Lashley, S., Barber, J., Chaytor, N., Miller, J. W., . . . Caylor, L. (2015). PACES in epilepsy: Results of a self-management randomized controlled trial. *Epilepsia, 56*(8), 1264-1274. doi:10.1111/epi.13052

Gandy, M., Heriseanu, A. I., Balakumar, T., Karin, E., Walker, J., Hathway, T., . . . Dear, B. F. (2023). The wellbeing neuro course: a randomised controlled trial of an internet-delivered transdiagnostic psychological intervention for adults with neurological disorders. *Psychological Medicine, 53*(14), 6817-6827. doi:10.1017/S0033291723000338

Gandy, M., Sharpe, L., Nicholson Perry, K., Thayer, Z., Miller, L., Boserio, J., & Mohamed, A. (2014). Cognitive behaviour therapy to improve mood in people with epilepsy: a randomised controlled trial. *Cogn Behav Ther, 43*(2), 153-166. doi:10.1080/16506073.2014.892530

Gold, S. M., Friede, T., Meyer, B., Moss-Morris, R., Hudson, J., Asseyer, S., . . . Heesen, C. (2023). Internet-delivered cognitive behavioural therapy programme to reduce depressive symptoms in patients with multiple sclerosis: a multicentre, randomised, controlled, phase 3 trial. *The Lancet Digital Health, 5*(10), e668-e678. doi:10.1016/S2589-7500(23)00109-7

Graziano, F., Calandri, E., Borghi, M., & Bonino, S. (2014). The effects of a group-based cognitive behavioral therapy on people with multiple sclerosis: a randomized controlled trial. *Clinical Rehabilitation, 28*(3), 264-274. doi:10.1177/0269215513501525

Hum, K. M., Chan, C. J., Gane, J., Conway, L., McAndrews, M. P., & Smith, M. L. (2019). Do distance-delivery group interventions improve depression in people with epilepsy? *Epilepsy & Behavior, 98*, 153-160. doi:10.1016/j.yebeh.2019.06.037

Johnson, E. K., Fraser, R. T., Lashley, S., Barber, J., Brandling-Bennett, E. M., Vossler, D. G., . . . Warheit-Niemi, T. (2020). Program of Active Consumer Engagement in Self-Management in Epilepsy: Replication and extension of a self-management randomized controlled trial. *Epilepsia, 61*(6), 1129-1141. doi:10.1111/epi.16530

Kirkness, C. J., Cain, K. C., Becker, K. J., Tirschwell, D. L., Buzaitis, A. M., Weisman, P. L., . . . Mitchell, P. H. (2017). Randomized trial of telephone versus in-person delivery of a brief psychosocial intervention in post-stroke depression. *BMC Res Notes, 10*(1), 500. doi:10.1186/s13104-017-2819-y

Kraepelien, M., Schibbye, R., Månsson, K., Sundström, C., Riggare, S., Andersson, G., . . . Kaldo, V. (2020). Individually Tailored Internet-Based Cognitive-Behavioral Therapy for Daily Functioning in Patients with Parkinson's Disease: A Randomized Controlled Trial. *J Parkinsons Dis, 10*(2), 653-664. doi:10.3233/jpd-191894

Lin, R. S. Y., Yu, D. S. F., Chau, P. H., & Li, P. W. C. (2023). Effects of an empowerment-based educative psycho-behavioral program on neuropsychiatric symptoms among persons with mild cognitive impairment: A mixed methods study. *Int J Nurs Stud, 137*, 104381. doi:10.1016/j.ijnurstu.2022.104381

Lincoln, N. B., Yuill, F., Holmes, J., Drummond, A. E., Constantinescu, C. S., Armstrong, S., & Phillips, C. (2011). Evaluation of an adjustment group forpeople with multiple sclerosis and lowmood: a randomized controlled trial. *Multiple Sclerosis Journal, 17*(10), 1250-1257. doi:10.1177/1352458511408753

Liu, Y. E., Lv, J., Sun, F. Z., Liang, J. J., Zhang, Y. Y., Chen, J., & Jiang, W. J. (2023). Effectiveness of group acceptance and commitment therapy in treating depression for acute stroke patients. *Brain Behav, 13*(12), e3260. doi:10.1002/brb3.3260

Majumdar, S., & Morris, R. (2019). Brief group-based acceptance and commitment therapy for stroke survivors. *British Journal of Clinical Psychology, 58*(1), 70-90. doi:10.1111/bjc.12198

Martin, P. R., Aiello, R., Gilson, K., Meadows, G., Milgrom, J., & Reece, J. (2015). Cognitive behavior therapy for comorbid migraine and/or tension-type headache and major depressive disorder: An exploratory randomized controlled trial. *Behaviour Research and Therapy, 73*, 8-18. doi:10.1016/j.brat.2015.07.005

Meyer, B., Weiss, M., Holtkamp, M., Arnold, S., Brückner, K., Schröder, J., . . . Nestoriuc, Y. (2019). Effects of an epilepsy-specific Internet intervention (Emyna) on depression: Results of the ENCODE randomized controlled trial. *Epilepsia, 60*(4), 656-668. doi:10.1111/epi.14673

Migliorini, C., Sinclair, A., Brown, D., Tonge, B., & New, P. (2016). A randomised control trial of an Internet-based cognitive behaviour treatment for mood disorder in adults with chronic spinal cord injury. *Spinal Cord, 54*(9), 695-701. doi:10.1038/sc.2015.221

Mohr, D. C., Hart, S. L., Julian, L., Catledge, C., Honos-Webb, L., Vella, L., & Tasch, E. T. (2005). Telephone-administered psychotherapy for depression. *Arch Gen Psychiatry, 62*(9), 1007-1014. doi:10.1001/archpsyc.62.9.1007

Moonen, A. J. H., Mulders, A. E. P., Defebvre, L., Duits, A., Flinois, B., Köhler, S., . . . Leentjens, A. F. G. (2021). Cognitive Behavioral Therapy for Anxiety in Parkinson's Disease: A Randomized Controlled Trial. *Mov Disord, 36*(11), 2539-2548. doi:10.1002/mds.28533

Nazari, N., Aligholipour, A., & Sadeghi, M. (2020). Transdiagnostic treatment of emotional disorders for women with multiple sclerosis: a randomized controlled trial. *BMC Women's Health, 20*(1), 245. doi:10.1186/s12905-020-01109-z

Nazari, N., Sadeghi, M., Ghadampour, E., & Mirzaeefar, D. (2020). Transdiagnostic treatment of emotional disorders in people with multiple sclerosis: randomized controlled trial. *BMC Psychology, 8*(1), 114. doi:10.1186/s40359-020-00480-8

Niu, Y., Sheng, S., Chen, Y., Ding, J., Li, H., Shi, S., . . . Ye, D. (2022). The Efficacy of Group Acceptance and Commitment Therapy for Preventing Post-Stroke Depression: A Randomized Controlled Trial. *J Stroke Cerebrovasc Dis, 31*(2), 106225. doi:10.1016/j.jstrokecerebrovasdis.2021.106225

Pahlavanzadeh, S., Abbasi, S., & Alimohammadi, N. (2017). The Effect of Group Cognitive Behavioral Therapy on Stress, Anxiety, and Depression of Women with Multiple Sclerosis. *Iran J Nurs Midwifery Res, 22*(4), 271-275. doi:10.4103/1735-9066.212987

Ponsford, J., Lee, N. K., Wong, D., McKay, A., Haines, K., Alway, Y., . . . O'Donnell, M. L. (2016). Efficacy of motivational interviewing and cognitive behavioral therapy for anxiety and depression symptoms following traumatic brain injury. *Psychol Med, 46*(5), 1079-1090. doi:10.1017/s0033291715002640

Potter, S. D. S., Brown, R. G., & Fleminger, S. (2016). Randomised, waiting list controlled trial of cognitive–behavioural therapy for persistent postconcussional symptoms after predominantly mild–moderate traumatic brain injury. *Journal of Neurology, Neurosurgery &amp; Psychiatry, 87*(10), 1075-1083. doi:10.1136/jnnp-2015-312838

Sadeghi-Bahmani, D., Esmaeili, L., Mokhtari, F., Sadeghi Bahmani, L., Afsharzadeh, M., Shaygannejad, V., . . . Gross, J. J. (2022). Effects of Acceptance and Commitment Therapy (ACT) and Mindfulness-Based Stress Reduction (MBSR) on symptoms and emotional competencies in individuals with multiple sclerosis. *Multiple Sclerosis and Related Disorders, 67*. doi:10.1016/j.msard.2022.104029

Schröder, J., Brückner, K., Fischer, A., Lindenau, M., Köther, U., Vettorazzi, E., & Moritz, S. (2014). Efficacy of a psychological online intervention for depression in people with epilepsy: a randomized controlled trial. *Epilepsia, 55*(12), 2069-2076. doi:10.1111/epi.12833

Simshäuser, K., Pohl, R., Behrens, P., Schultz, C., Lahmann, C., & Schmidt, S. (2022). Mindfulness-Based Cognitive Therapy as Migraine Intervention: a Randomized Waitlist Controlled Trial. *International Journal of Behavioral Medicine, 29*(5), 597-609. doi:10.1007/s12529-021-10044-8

Spruill, T. M., Friedman, D., Diaz, L., Butler, M. J., Goldfeld, K. S., O'Kula, S., . . . Devinsky, O. (2021). Telephone-based depression self-management in Hispanic adults with epilepsy: a pilot randomized controlled trial. *Transl Behav Med, 11*(7), 1451-1460. doi:10.1093/tbm/ibab045

Sun, Q., Xu, H., Zhang, W., Zhou, Y., & Lv, Y. (2022). Behavioral Activation Therapy for Subthreshold Depression in Stroke Patients: An Exploratory Randomized Controlled Trial. *Neuropsychiatr Dis Treat, 18*, 2795-2805. doi:10.2147/ndt.S392403

Thomas, S. A., Drummond, A. E., Lincoln, N. B., Palmer, R. L., das Nair, R., Latimer, N. R., . . . Topcu, G. (2019). Behavioural activation therapy for post-stroke depression: the BEADS feasibility RCT. *Health Technol Assess, 23*(47), 1-176. doi:10.3310/hta23470

Thomas, S. A., Walker, M. F., Macniven, J. A., Haworth, H., & Lincoln, N. B. (2012). Communication and Low Mood (CALM): a randomized controlled trial of behavioural therapy for stroke patients with aphasia. *Clin Rehabil, 27*(5), 398-408. doi:10.1177/0269215512462227

Visser, M. M., Heijenbrok-Kal, M. H., Spijker, A. v. t., Lannoo, E., Busschbach, J. J. V., & Ribbers, G. M. (2016). Problem-Solving Therapy During Outpatient Stroke Rehabilitation Improves Coping and Health-Related Quality of Life. *Stroke, 47*(1), 135-142. doi:doi:10.1161/STROKEAHA.115.010961

Wang, X., Li, J., Wang, C., & Lv, J. (2020). The effects of mindfulness-based intervention on quality of life and poststroke depression in patients with spontaneous intracerebral hemorrhage in China. *International Journal of Geriatric Psychiatry, 35*(5), 572-580. doi:<https://doi.org/10.1002/gps.5273>
